# Supplementary material for: Timing of Novel Drug 1A-116 to Circadian Rhythms Improves Therapeutic Effects against Glioblastoma
Source: Pharmaceutics. 2021 Jul 16;13(7):1091. doi: 10.3390/pharmaceutics13071091 (PMC8309043; doi:10.3390/pharmaceutics13071091)
Supplement: Supplementary file 1 [file pharmaceutics-13-01091-s001.zip › pharmaceutics-1279948-supplementary.pdf]

pharmaceutics

MDPI

Supplementary Materials: Timing of Novel Drug 1A-116 to Circadian Rhythms Improves Therapeutic Effects against Glioblastoma

Laura Lucía Trebucq, Georgina Alexandra Cardama, Pablo Lorenzano Menna, Diego Andrés Golombek, Juan José Chiesa and Luciano Marpegan

Primers for exon 1, 2 and 3 of *p53* gene to detect Pro98Leu mutation, and primers for exon 1, 2, 3 and 4 of *PTN* gene were designed. Oligos for *cyclin dependent kinase inhibitor 2A*, that encodes for many transcriptional variants, including *p14ARF* and *p16* genes, were also designed.

<u>P53</u>

```
Alignment:   Global DNA alignment against reference molecule
Parameters:  Scoring matrix:  Linear (Mismatch 2, OpenGap 4, ExtGap 1)

   Reference molecule:  mRNA p53var1, Region 1 to 2591
   Number of sequences to align:  3
   Total length of aligned sequences with gaps:  2591 bps
   Settings:  Similarity significance value cutoff:  >= 60%


Summary of Percent Matches:
   Ref:  mRNA p53var1          1 to   2591   (  2591 bps)      --
     2:  ex3p53                1 to    253   (   253 bps)       9%
     3:  ex1p53                1 to     67   (    67 bps)       2%


mRNA p53var1       1 gatgggattggggttttcccctcccatgtgctcaagactggcgctaaaagttttgagctt
ex3p53               ------------------------------------------------------------
ex1p53               ------------------------------------------------------------

mRNA p53var1      61 ctcaaaagtctagagccaccgtccagggagcaggtagctgctgggctccggggacacttt
ex3p53               ------------------------------------------------------------
ex1p53               ------------------------------------------------------------

mRNA p53var1     121 gcgttcgggctgggagcgtgctttccacgacggtgacacgcttccctggattggcagcca
ex3p53               ------------------------------------------------------------
ex1p53               ------------------------------------------------------------

mRNA p53var1     181 gactgccttccgggtcactgccatggaggagccgcagtcagatcctagcgtcgagccccc Ex1
ex3p53               ------------------------------------------------------------
ex1p53             1 ----------------------atggaggagccgcagtcagatcctagcgtcgagccccc

mRNA p53var1     241 tctgagtcaggaaacattttcagacctatggaaactacttcctgaaaacaacgttctgtc Ex2
ex3p53               ------------------------------------------------------------
ex1p53            39 tctgagtcaggaaacattttcagacctat-------------------------------

mRNA p53var1     301 ccccttgccgtcccaagcaatggatgatttgatgctgtccccggacgatattgaacaatg Ex3
ex3p53             1 ----------------------gatgatttgatgctgtccccggacgatattgaacaatg
ex1p53               ------------------------------------------------------------

mRNA p53var1     361 gttcactgaagacccaggtccagatgaagctcccagaatgccagaggctgctccccccgt Ex3
ex3p53            39 gttcactgaagacccaggtccagatgaagctcccagaatgccagaggctgctccccccgt
ex1p53               ------------------------------------------------------------

mRNA p53var1     421 ggcccctgcaccagcagctcctacaccggcggcccctgcaccagccccctcctggcccct Ex3
ex3p53            99 ggcccctgcaccagcagctcctacaccggcggcccctgcaccagccccctcctggcccct
ex1p53               ------------------------------------------------------------

mRNA p53var1     481 gtcatcttctgtccctTcccagaaaacctaccagggcagctacggtttccgtctgggctt Ex3
ex3p53           159 gtcatcttctgtccttTcccagaaaacctaccagggcagctacggtttccgtctgggctt
ex1p53               ------------------------------------------------------------

mRNA p53var1     541 cttgcattctgggacagccaagtctgtgacttgcacgtactcccctgccctcaacaagat Ex3
ex3p53           219 cttgcattctgggacagccaagtctgtgacttgca-------------------------
ex1p53               ------------------------------------------------------------

mRNA p53var1     601 gttttgccaactggccaagacctgccctgtgcagctgtgggttgattccacacccccgcc
ex3p53               ------------------------------------------------------------
ex1p53               ------------------------------------------------------------
```

In red, mutated codon 98 CCT-CTT.

PTN

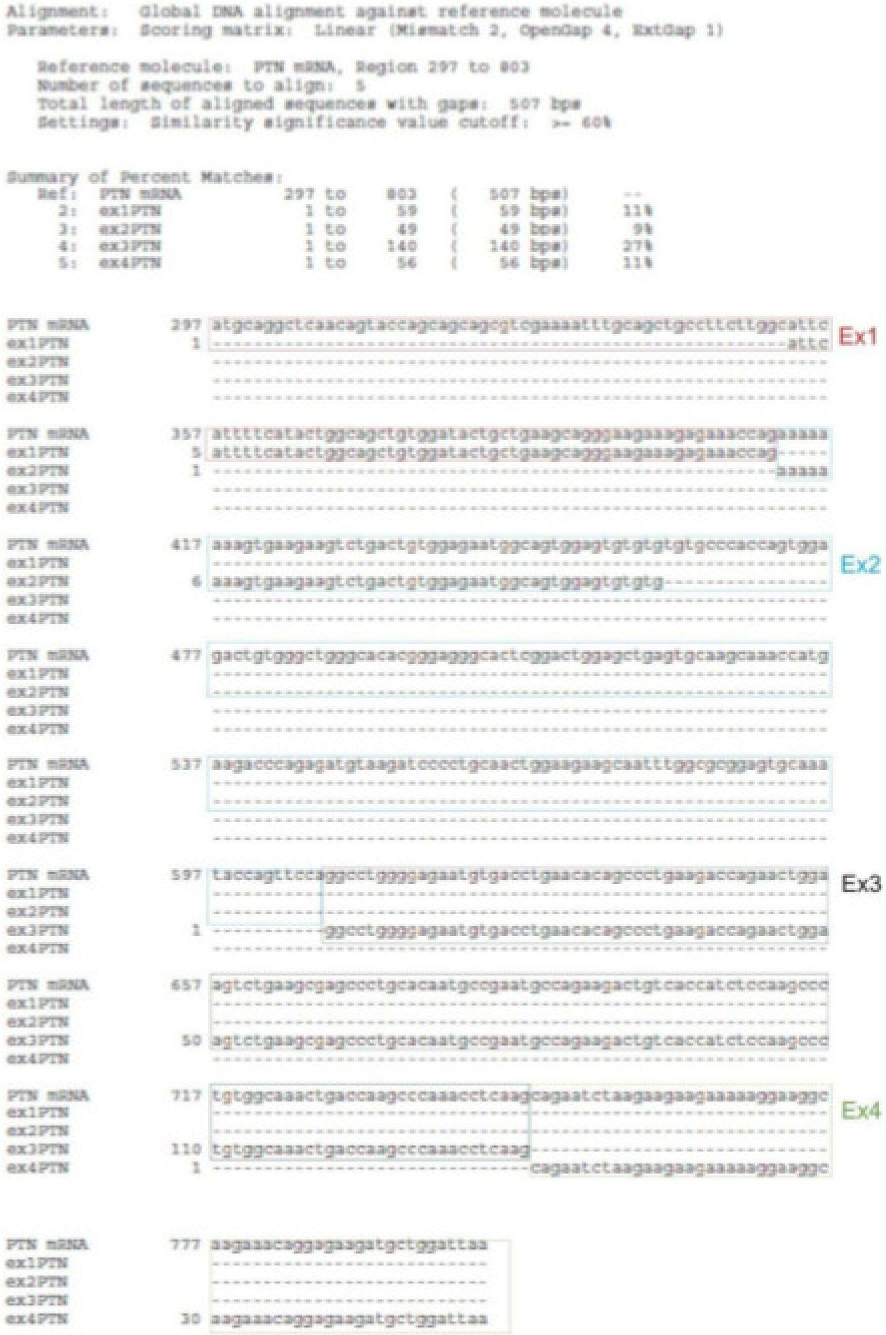

P14 and p16

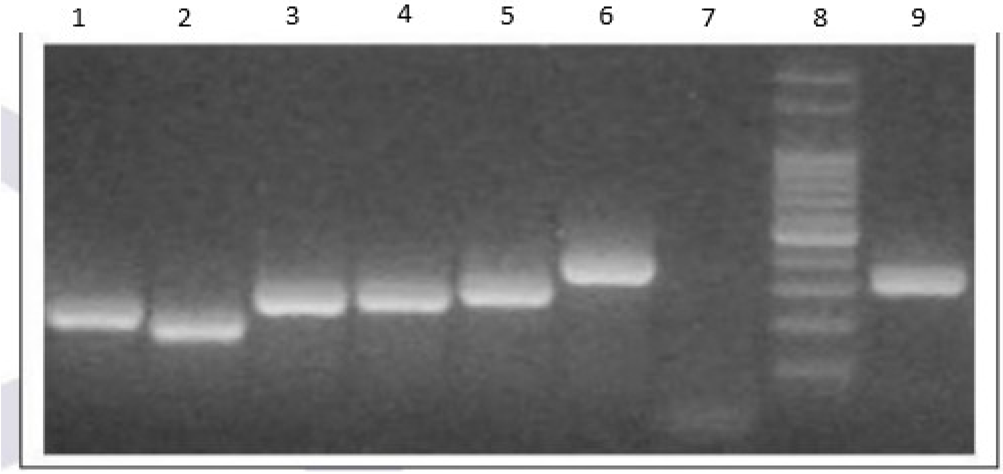

1: Exon 4 PTN 2: Exon 1 PTN 3: Exon 3 PTN 4: Exon 2 PTN 5: Ex1-Ex2 – P53
6: Ex3 –P53 **7: p16/p14ARF** 8: Ladder 100 pb 9: Human distrofin gene fragment (Ctrol +)

Genetic profile of LN229 cell line evaluated by STR.

**Amelogenina**	X
**D3S1358**	16, 17
**D1S1656**	14, 17
**D2S441**	10, 11
**D10S1248**	16
**D13S317**	10, 11
**PENTA E**	7, 16
**D16S539**	12
**D18S51**	13, 15
**D2S1338**	19, 20
**CSF1PO**	12
**PENTA D**	10, 11
**TH01**	9.3
**VWA**	16, 19
**D21S11**	29, 30
**D7S820**	8, 11
**D5S818**	11
**TPOX**	8
**D8S1179**	13, 14
**D12S391**	20, 22
**D19S433**	12, 17.2
**SE33**	29.2
**D22S1045**	15, 16
**FGA**	23
**DYS391**	NA
**DYS576**	NA

Figure S1. LN229 cell line authentication.

Sequence alignment performed with Blast, Query: sequence corresponding to a fragment of exon 2 of *bmal1*, Subject: sequence corresponding to *bmal1* WT. In red: site of Crispr/Cas9 editing. Identity: 254/256 (99%).

```
Sequence ID: Query_63447 Length: 320
Range 1: 39 to 293

Score:460 bits(249), Expect:1e-134,
Identities:254/256(99%),  Gaps:2/256(0%), Strand: Plus/Plus

Query  5    ACAGTT-CATGGGTACTGAGAGGAGGGGGTGCCTGGCCTGCCTGGAACACTGGGGAGGCT  63
            |||||| |||||||||||||||||||||||||||||||||||||||||||||||||||||
Sbjct  39   ACAGTTCCATGGGTACTGAGAGGAGGGGGTGCCTGGCCTGCCTGGAACACTGGGGAGGCT  98

Query  64   TACAGGGAAAAACTGTCCAATGGATTTAAAGGACAAGCAGGGATTTTCTAGGCAAAGAAG  123
            ||||||||||||||||||||||||||||||||||||||||||||||||||||||||||||
Sbjct  99   TACAGGGAAAAACTGTCCAATGGATTTAAAGGACAAGCAGGGATTTTCTAGGCAAAGAAG  158

Query  124  CAGAATGGGCATCCCAGGAGTATGGTAAATCAAAACTTATTGGGTGCTATGAATTTAAAG  183
            ||||||||||||||||||||||||||||||||||||||||||||||||||||||||||||
Sbjct  159  CAGAATGGGCATCCCAGGAGTATGGTAAATCAAAACTTATTGGGTGCTATGAATTTAAAG  218

Query  184  CTAAACTTTTATTTCATTGGCTTTTAGGTGGTGCTGGCTAGAGTGTATACCGTTTGGACC  243
            ||||||||||||||||||||||||||||||||||||||||||||||||| ||||||||||
Sbjct  219  CTAAACTTTTATTTCATTGGCTTTTAGGTGGTGCTGGCTAGAGTGTATA-CGTTTGGACC  277

Query  244  CAAGCTTAACTTTTCC  259
            ||||||||||||||||
Sbjct  278  CAAGCTTAACTTTTCC  293
```

Sequence alignment performed with Blast, Query: sequence corresponding to a fragment of exon 2 of *bmal1*, Subject: sequence corresponding to *bmal1* E1. In red: site of Crispr/Cas9 editing. Identity: 256/262 (98%)

```
Sequence ID: Query_12599 Length: 320
Range 1: 39 to 299

Score:449 bits(243), Expect:3e-131,
Identities:256/262(98%),  Gaps:2/262(0%), Strand: Plus/Plus

Query  7    ACAGTT-CATGGGTACTGAGAGGAGGGGGTGCCTGGCCTGCCTGGAACACTGGGGAGGCT  65
            |||||| |||||||||||||||||||||||||||||||||||||||||||||||||||||
Sbjct  39   ACAGTTCCATGGGTACTGAGAGGAGGGGGTGCCTGGCCTGCCTGGAACACTGGGGAGGCT  98

Query  66   TACAGGGAAAAACTGTCCAATGGATTTAAAGGACAAGCAGGGATTTTCTAGGCAAAGAAG  125
            ||||||||||||||||||||||||||||||||||||||||||||||||||||||||||||
Sbjct  99   TACAGGGAAAAACTGTCCAATGGATTTAAAGGACAAGCAGGGATTTTCTAGGCAAAGAAG  158

Query  126  CAGAATGGGCATCCCAGGAGTATGGTAAATCAAAACTTATTGGGTGCTATGAATTTAAAG  185
            ||||||||||||||||||||||||||||||||||||||||||||||||||||||||||||
Sbjct  159  CAGAATGGGCATCCCAGGAGTATGGTAAATCAAAACTTATTGGGTGCTATGAATTTAAAG  218

Query  186  CTAAACTTTTATTTCATTGGCTTTTAGGTGGTGCTGGCTAGAGTGTATACCGATTGGACC  245
            ||||||||||||||||||||||||||||||||||||||||||||||||| || |||||||
Sbjct  219  CTAAACTTTTATTTCATTGGCTTTTAGGTGGTGCTGGCTAGAGTGTATA-CGTTTGGACC  277

Query  246  CAAGCTTATCTTTTGCGATGTG  267
            |||||||| ||||| | |||||
Sbjct  278  CAAGCTTAACTTTTCCAATGTG  299
```

Figure S2. Confirmation of genomic edition of Exon2 of bmal1 by Crispr/Casp9.

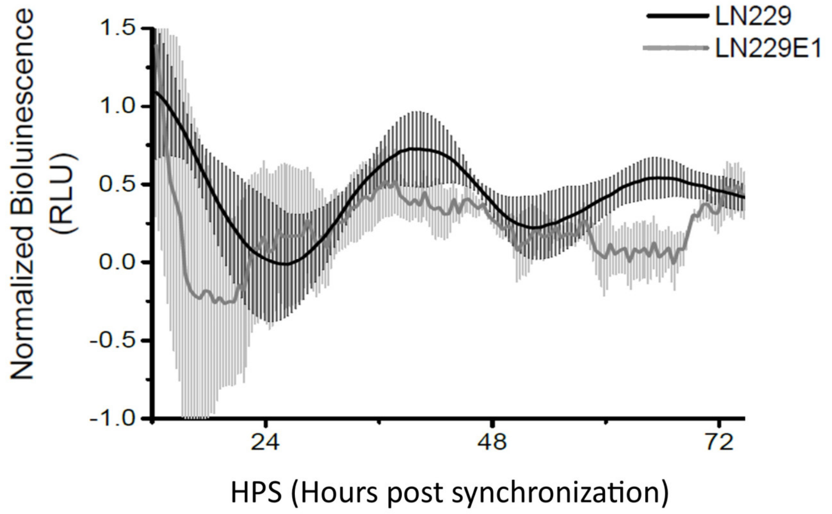

Figure S3. Circadian expression of Bmal1 is maintained for over 72 h.

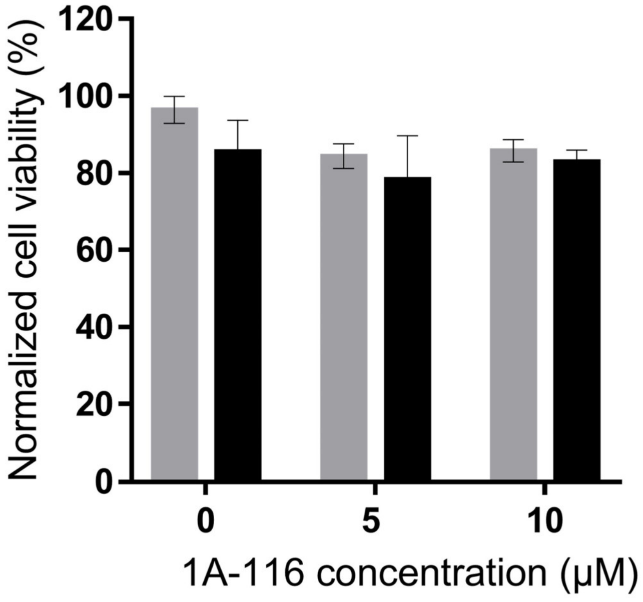

Figure S4. Cell viability.

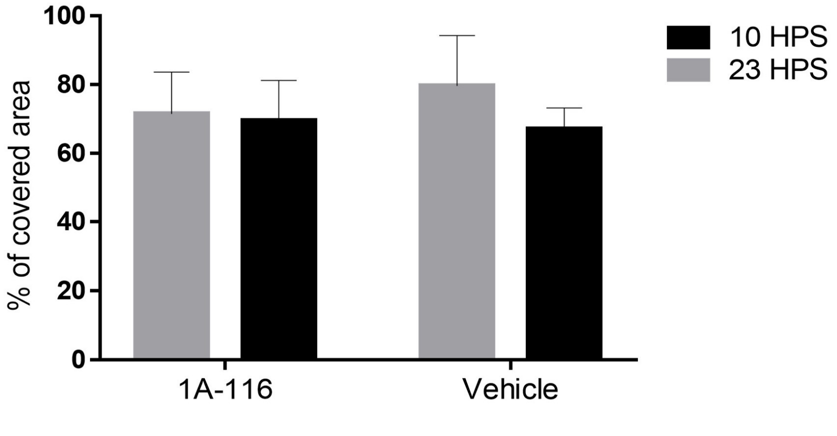

Figure S5. Number of cells outside of the migration area.
